# Movie Review Summarization Using Supervised Learning and Graph-Based Ranking Algorithm

**DOI:** 10.1155/2020/7526580

**Published:** 2020-06-02

**Authors:** Atif Khan, Muhammad Adnan Gul, Mahdi Zareei, R. R. Biswal, Asim Zeb, Muhammad Naeem, Yousaf Saeed, Naomie Salim

**Affiliations:** ^1^Department of Computer Science, Islamia College University Peshawar, Peshawar 25000, KP, Pakistan; ^2^Tecnologico de Monterrey, Escuela de Ingenieria y Ciencias, Zapopan, Jalisco 45138, Mexico; ^3^Department of Computer Science, Abbottabad University of Science and Technology, Abbottabad 25000, Pakistan; ^4^Department of Information Technology, University of Haripur, Haripur, KP, Pakistan; ^5^School of Computing, Faculty of Engineering, Universiti Teknologi Malaysia, Johor Bahru, Malaysia

## Abstract

With the growing information on web, online movie review is becoming a significant information resource for Internet users. However, online users post thousands of movie reviews on daily basis and it is hard for them to manually summarize the reviews. Movie review mining and summarization is one of the challenging tasks in natural language processing. Therefore, an automatic approach is desirable to summarize the lengthy movie reviews, and it will allow users to quickly recognize the positive and negative aspects of a movie. This study employs a feature extraction technique called bag of words (BoW) to extract features from movie reviews and represent the reviews as a vector space model or feature vector. The next phase uses Naïve Bayes machine learning algorithm to classify the movie reviews (represented as feature vector) into positive and negative. Next, an undirected weighted graph is constructed from the pairwise semantic similarities between classified review sentences in such a way that the graph nodes represent review sentences, while the edges of graph indicate semantic similarity weight. The weighted graph-based ranking algorithm (WGRA) is applied to compute the rank score for each review sentence in the graph. Finally, the top ranked sentences (graph nodes) are chosen based on highest rank scores to produce the extractive summary. Experimental results reveal that the proposed approach is superior to other state-of-the-art approaches.

## 1. Introduction

With the development of Web 2.0 that emphasizes the participation of users, more and more websites such as Internet Movie Database (IMBD, a movie review website) and Amazon encourage users to post reviews for the products they are interested in. In order to satisfy customers and enhance their shopping experience, online merchants often ask their customers to give opinions or reviews on the products or services they purchased online. The number of reviews received by a product grows rapidly as millions of customers post reviews about a product, which results in information overload [1]. Due to this information overload, it is difficult for a customer to scan each review of a product in order to make a decision whether to purchase a product or not. At the same time, it is also hard for online merchants/product manufacturer or service provider to keep track of huge amount of reviews posted by customers about their products or services [1]. In order to overcome this problem, there is a need for automatic review mining and summarization system [2].

In this paper, we will focus on the movie review domain. Considering the movie, summarizing thousands of reviews received by a movie can help the viewer (customer) to swiftly scan the summary of it and quickly decide whether to watch a movie or not. On the other hand, the summary of movie reviews can assist the movie service provider such as Netflix to swiftly understand the watching patterns or the interests of their customers.

This study proposes an automatic approach to mine and summarize the movie reviews. Such approach will assist the new user to swiftly understand the negative and positive aspects of a movie, and hence the user can quickly decide whether to watch a movie or not. The task of review mining and summarization (RMS) comprises of two steps: the first step is review mining, which mines the reviews received by a movie by classifying them into positive and negative. The second step is review summarization which generates a concise summary from the classified reviews.

Nowadays, RMS gained significant attention in many areas [3]. For example, from the online reviews of political news or announcements, the government can perceive the influence of recent policies (or events) on common people and take proper and timely actions based on the information. Product reviews, on other hand, collect feedback from customers, and summarizing such customer feedback assists the online manufacturer/retailer to know about their products perceived by the customers.

Review mining or sentiment analysis [4] classifies the review text into positive or negative. There are various approaches to classify user review text into positive and negative review such as machine learning (ML) approaches and dictionary-based approaches. Many ML-based approaches such as Naïve Bayes (NB) [5], decision tree [6], support vector machine (SVM) [7], and neural networks [8] have been presented for text classification and revealed their capabilities in various domains. NB is one of the state-of-the-art algorithms and has been proved to be highly effective in traditional text classification. The classification decisions of NB are remarkably good. NB is frequently used as a baseline in text classification and sentiment analysis research since it combines good accuracy with efficiency [8, 9]. Therefore, this study employs NB for movie review classification. On the other hand, dictionary-based approaches use word lexicons for semantic orientation of document [10]. However, dictionary-based approaches are incapable to deal with domain-specific orientations.

Review summarization is the process of generating summary from gigantic reviews sentences [11]. Numerous techniques for review summarization such as supervised ML-based techniques [6, 7] unsupervised/lexicon-based techniques [6, 12–16] have been applied. However, the unsupervised/lexicon-based approaches heavily rely on linguistic resources and are limited to words present in the lexicon. On the other hand, mostly supervised ML approaches performed better than unsupervised ML-based approaches but they are applied in specific domains. Previous research shows that text summarization has been successfully applied in numerous domains [7, 17–21]. The text summarization technique is employed to extract the salient information from source text and produce a condensed version of the text for different users [22–25]. For instance, given a user query, the Google search can give several website links along with the short summary concerning the content of each website, which assists users to decide whether the websites are useful or not. Example of famous application software for text summarization is Summly, which can automatically extract significant news articles and then display the summary of each news article based on news categories chosen by users.

Several users post bulk reviews on movie review websites such as IMDB on daily basis, which involve user attitude towards a specific movie. Thus, automatically mining and summarizing these bulk reviews is desirable. However, the previous approaches proposed for movie summarization are limited to generate feature-based summary rather than generic summary. Therefore, this study proposes a review mining and summarization (RMS) approach that integrates supervised ML approach with graph-based ranking algorithm to automatically generate a generic summary of movie reviews. The proposed approach operates in the following manner: first, we employ a simple feature extraction technique called bag of words (BoW) to extract features from movie reviews and represent them as a vector space model or feature vector. The next phase uses Naïve Bayes classifier to classify the movie reviews into positive and negative. Next, the classified reviews are segmented into sentences and then we use word2vec model to extract word embeddings for each word in sentences. The sentence embeddings/vectors are formed by taking mean of all word embeddings in sentences. The pairwise semantic similarities between sentences are computed by taking cosine similarity of corresponding sentence embeddings. Next, an undirected weighted graph is constructed from the pairwise semantic similarities between classified review sentences in such a way that the graph nodes represent review sentences, while the edges of graph indicate semantic similarity weight. The weighted graph-based ranking algorithm (WGRA) is applied to compute the rank score for each review sentence in the graph. Finally, the top ranked sentences (graph nodes) are chosen based on highest rank scores to produce the extractive summary. Our contributions are summarized as follows:

- To classify the movie reviews by using Naïve Bayes machine learning algorithm with both unigrams and bigrams as feature set.
- To propose a graph-based ranking algorithm embedded with semantic similarity to produce a generic extractive summary from classified movie reviews.
- To evaluate the proposed summarization approach with the state-of-the-art approaches in context of ROUGE-1 and ROUGE-2 evaluation metrics.

The rest of this paper is organized as follows.  Section 2 demonstrates the related work to this research.  Section 3 outlines the proposed approach.  Section 4 presents the evaluation results and discussion. Finally, we end with conclusion along with the future work in Section 5.

## 2. Related Work

The task of review mining and summarization consists of two major steps: review mining and review summarization. First, we discuss the relevant literature to review mining followed by review summarization. Review mining or opinion mining is the process of extracting, analysing, and classifying subjective information and determining the sentiment that is associated with a particular target. Different methods are proposed for the task of review mining by many researchers [4]. For example, considering a document of review text *A* = [*a*_1_, *a*_2_, *a*_3_,…, *a*_*n*_] with a category set *B* = [Positive, Negative], the job of review mining is to mine each review sentence *a*_*i*_ in the document A, with a predefined category label (Positive or Negative) in set B [26].

Numerous review mining approaches such as ML-based and sentiment lexicon-based techniques have been proposed for mining reviews in different domains [1, 7, 27, 28]. The authors in [29] presented applications and challenges in the area of review/opinion mining. ML-based algorithms [5, 29, 30] are also utilized for opinion classifications of documents. The ML algorithms are classified into two categories: supervised and unsupervised ML techniques. These techniques achieve the goal of sentiment classification on the basis of extraction and selection of set of appropriate features.

Supervised machine learning technique such as SVM [7] is applied for sentiment classification of movie review data. The authors in [6] used decision tress to classify high/low informative opinion phrases extracted from restaurant reviews. On the other hand, the authors in [12] used unsupervised ML techniques such as unsupervised feature clustering with latent Dirichlet allocation (LDA) for obtaining labeled features. They trained the initial sentiment classifier with prior information (labeled features) from sentiment lexicon. The sentiment classifier learnt from unlabeled review documents extracted from different domains such as movies, books, and electronics. The labeled features were then used for the model's predictions on unlabeled instances using generalized expectation (GE) criteria. The authors in [13] introduced the OPINE system, which determined the semantic orientation of words by using relaxation labeling. A pulse system [14] mined topics and sentiment orientation from customer feedback sampled from car reviews database. The system trained a sentiment classifier by using a bootstrapping process.

Next, we discuss sentiment lexicon-based approach for review mining, which can be classified in two categories: dictionary-based [15] and corpus-based [16] approaches. The authors in [15] proposed a dictionary-based approach integrated with WordNet graph for polarity classification. The approach determined polarity scores from thesaurus such as SentiWordNet [10] and combined it with random walk analysis of concepts found in the movie reviews. The dictionary-based approach suffered from a limitation that it is incapable of dealing with context and domain-specific orientation since same term might have different meanings in different domains. The authors in [16] proposed a corpus-based approach by utilizing a corpus of movie reviews that are annotated manually. The approach obtained linguistic features such as nouns, verbs, adjectives, and adverbs by performing Parts-of-Speech (POS) tagging over the movie reviews. They also exploited a semantic resource such as SentiWordNet for computing the polarity score of movie review document in the corpus. Both corpus- and dictionary-based approaches heavily rely on linguistic resources and are limited to words present in the lexicon.

Numerous techniques for review summarization have also been explored. Review summarization is an important step in the task of review mining and summarization [4], which extracts salient information from the review text and presents them in the form of a summary. The final summary might be feature-based summary or it can be generic summary covering the general information about a product (camera, cellular phone, and movie) in a concise manner [4]. The authors in [1] proposed an approach for feature-based summary for customer product (camera and cellular phone) reviews. The approach used word attributes, including Parts of Speech (POS), occurrence frequency, and synset in WordNet. The final summary was organized according to extracted features. The authors in [7] introduced a latent-semantic analysis- (LSA-) based approach to identify product features from movie reviews. In order to generate a review summary, opinion words and product features were used to choose relevant sentences to form a review summary. However, this approach was limited to Chinese movie reviews and has not been applied to English movie reviews. A multiknowledge approach was proposed in [3] for movie review summarization. The approach utilized WordNet and labeled movie training data and movie casts to produce a keyword list for determining features and opinions. Finally, summary sentences are reorganized according to the extracted features. However, this approach might not be able to find valid feature-opinion pairs as grammatical relations do not check the semantic relationship between opinion words and features. However, the previous approaches proposed for movie summarization are limited to generate feature-based summary rather than generic summary. Therefore, we proposed a text summarization approach based on supervised ML integrated with graph-based ranking algorithm to produce a generic summary of movie reviews. Moreover, a text summarization approach based on unsupervised ML [31] has also been proposed to generate summary from online hotel reviews. However, this approach is limited to hotel reviews. On the other hand, we proposed a supervised ML approach for a different domain such as movie reviews.

Text summarization techniques have been employed in different application domains, such as summaries of webpages, patents, and news articles [32, 33]. The authors in [34] presented a text summarization technique to produce summaries from patents. The approach used different features such as position of sentence and cue phrases while determining the importance of sentence. The authors in [35] applied term TF-IDF technique and ontology tree structure techniques for finding keywords and extracting the important content of a patent document. The important sentences were then grouped using a clustering technique to produce a summary. The authors in [36] generated summaries from webpages. The approach performed a query expansion by using WordNet and then the expanded query is given to the Google search engine to find related documents. The final summary is produced based on sentences containing the relevant keywords. A statistical method was proposed in [37] for news article summarization. The sentences in the news documents are scored based on different features such length of sentence, first sentence of news article, title of news article, proper nouns, and term frequency. The top scored sentences are selected to produce a summary. The authors in [38] presented a pattern-based method for news article summarization.

In recent years, various graph-based methods have attracted more attention and effectively attempted for text summarization. These methods utilize PageRank algorithm [39] and its variants to give rank/score to graph nodes, which represent sentences or passages. The authors in [40] proposed a connectivity graph, which assumes that nodes only carry significant information if they are connected to many other nodes. The authors in [41] introduced a Lex-PageRank approach that is based on eigenvector centrality, which constructs a sentence connectivity matrix and utilizes similar algorithm like PageRank to find the significant sentences for summary. A similar algorithm to PageRank was also proposed in [42] which finds salient sentences for summary generation. The authors in [24] presented a graph-based approach, which integrates surface features with text content and investigates subtopic features in multiple documents to incorporate them into the graph-based ranking algorithm. A multidocument summarization approach based on affinity graph [43] exploits similar algorithm to PageRank and computes sentence scores in the affinity graph based on information richness. The authors in [44] demonstrated a document-sensitive graph model for multidocument generic summarization and highlighted the impact of global document set information at sentence level. A weighted graph model for generic multidocument summarization introduced in [45] combines sentence ranking and sentence clustering methods. The authors in [46] presented a graph-based method for multidocument summarization of Vietnamese documents and employed traditional PageRank algorithm to rank the important sentences. The authors in [47] demonstrated an event graph-based approach for multidocument extractive summarization. However, the approach requires the construction of hand crafted rules for argument extraction, which is a time consuming process and may limit its application to a specific domain.

All the previous graph-based summarization approaches were applied to new articles domain and employed a simple PageRank algorithm. However, we propose a graph-based summarization approach for movie review domain and employ a weighted graph-based ranking algorithm embedded with semantic similarity.

Recent research studies are exploiting the capabilities of deep learning and reinforcement learning approaches [48–51] to improve the text summarization task. The prevalent challenge in applying deep learning and reinforcement learning for text summarization is the unavailability of manually created extractive summaries that are required as ground truth for training the networks. The authors in [52] also presented a comprehensive survey on extractive and abstractive techniques for text summarization. The detail of our proposed approach is presented in the next section.

## 3. Proposed Methodology

**Figure 1:**
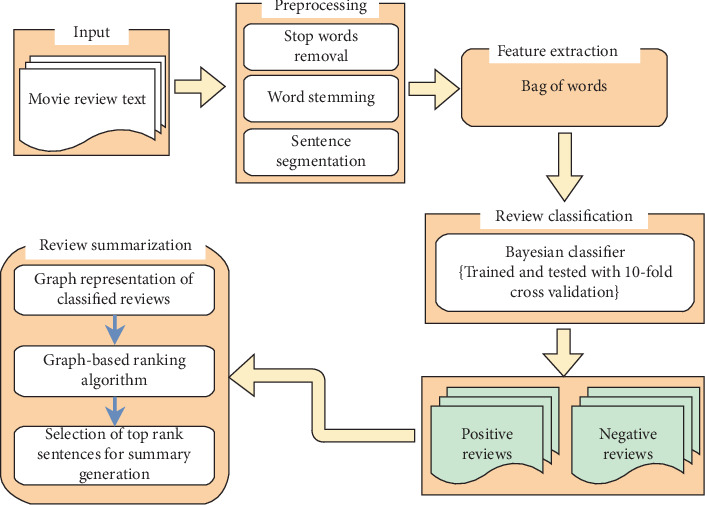
Proposed approach for movie reviews classification and summarization.

In this section, research framework of the proposed study is presented.  Figure 1 depicts the proposed framework. The framework is divided into four phases: (1) preprocessing, (2) feature extraction, (3) classification of reviews, and (4) summarization of reviews.

### 3.1. Preprocessing

The preprocessing of data in computational linguistic is a significant procedure, particularly in review mining and summarization (RMS). As the suggested work is related to RMS, the review document needs to be preprocessed so that it can be used in experiment efficiently before giving it as an input to the system. The preprocessing phase involves four steps, i.e., sentence segmentation, tokenization, stop words removal, and word stemming.

(a) Sentence segmentation: it is an essential step in NLP applications such as IR, machine translation, semantic role labeling, and summarization. It is the process of boundary detection within a document which splits the document text into sentences. Mostly, full stop/period (.), sign of exclamation (!), or a sign of interrogation (?) is commonly used to signify boundary of a sentence [53].
For example, we have a text document: “I like this movie. It is one of the best movies.”
After segmentation of the above text document we get a string list.
    Input review text:
    “I like this movie. It is one of the best movies.”
    Output: segmented text:
    Segment 1: “I like this movie.”
    Segment 2: “It is one of the best movies.”
(b) Tokenization: in this task, we use a simple program to split the sentences into distinct words by splitting them at whitespaces such as blanks, tabs, and punctuation marks such as period, semicolon, comma, and colon which are the primary cues for splitting the text into tokens.
(c) Stop words removal: words that appear frequently in the document are called stop words. It consists of conjunctions, articles, prepositions, and frequent words like “the,” “I,” “an,” and “a”. Stop words carry very little or no meaning in the document, so it is a good idea to remove them from document set. Eliminating stop words from review documents helps to increase the performance of the system. Buckley stop word list [54] is employed in the proposed framework.
(d) Word stemming: it is an important task in the preprocessing phase. Word stemming transforms the derived words to its stem or root word for capturing the similar concept. In this study, a well-known stemming algorithm named as Porter's stemming [55] is used for word stemming that removes the suffixes of words. For example, the words “watching,” “watches,” and “watchers” will be transformed to its root word “watch” with the help of stemming algorithm by removing suffixes -ing, -es, and -ers. The proposed method used this step to select meaningful words from the review sentences.

### 3.2. Feature Extraction

The aim of this phase is to extract features for review classification by employing a well-known feature extraction technique called bag of words (BoW). BoW is a simple feature extraction technique that represents the review text document as a vector space model. Each dimension of a vector space represents a feature. In this study, we use both unigrams and bigrams as feature set. The features in the vector space represent all the possible unigrams and bigrams (two word sequence) from the review text document, whereas the values of features refer to frequency or occurrence of unigrams/bigrams contained in the review text document. The BoW approach represents each document as a bag of words (unigrams) ignoring the grammar and order of words in a text document.

**Example 1 .**

Consider the following three review text documents, and for the sake of convenience, we have shown a single review sentence from each document.

  Review document 1: “I loved this movie.”
  Review document 2: “I hated this movie.”
  Review document 3: “Great acting a good movie.”

There are 7 unique words (unigrams) extracted from the above review sentences. The extracted unigrams refer to the features which are “Acting,” “good,” “Great,” “hated,” “Loved,” “Movie,” and “this.” The collection of features representing the review text documents represents the vector space model. The values of features in Table 1 indicate the frequencies of unigrams.

In order to boost the sentiment classification accuracy; this study combines unigrams with bigrams (*two-word pair*) vector space representation of a review. Bag of bigram refers to two-word pair in computational linguistics, for instance, “great movie,” “beautiful sky,” “not yet,” etc. Bigrams such as “good job,” “well done,” and “Pretty good” have positive orientation. On the other hand, bigrams like “quite expensive,” “no good,” and “bad luck” have negative orientation and bigrams like “to be” has neutral orientation.

On the other hand, BoW (unigram) approach splits a two-word pair such as “no good” into “no” and “good,” and hence the word “good” is considered as positive oriented. Bigrams also help to reduce vector space dimensions.  Table 2 depicts bag of bigram vector space model representation for the review documents. Referring to Example 1, bag of bigram vector space model for the review documents is shown below.

Table 3 shows the vector space model representation of bag of unigrams and bigrams for the review documents given in Example 1.

### 3.3. Classification of Reviews

The goal of this phase is to classify users' review text using supervised ML classification algorithm. The task of review classification categorizes the user's reviews into positive and negative. In this study, we have used Naïve Bayes (NB) classification algorithm since it is a robust classifier [56] and achieved higher accuracy on scalable datasets as compared to other state-of-the-art classification algorithms. Moreover, NB classifier has several applications in text classification because of its simplicity and accuracy [56].

In order to classify the reviews, the feature vectors along with their labels are given as input to the classifier. Probability of a term's given certain category (positive or negative) is calculated based on number of times a term occurs with that category in the review documents. Here, the term refers to either unigram or bigram or trigram since the features used in this study are both unigrams and bigrams. In order to classify a new review document, the probability of each term (unigram, bigram, and trigram) in the document's given class label (+ve) is determined, and then the probability of review document's given class label (+ve) is calculated by multiplying the probabilities of all terms with the probability of target class (+ve). Similarly, the probability of review document's given class label (−ve) is calculated.

The review document is classified as positive if its probability of given target class (+ve) is maximized; otherwise, it is classified as negative.

Bayes' theorem is stated mathematically as follows:

(1) $$\begin{array}{c} P\left(\left.A\right|B\right)=\frac{P\left(\left.B\right|A\right)P\left(A\right)}{P\left(B\right)}. \end{array}$$

Consider a new review document “*I love this movie*” is given to the NB classifier which will classify it into either positive or negative. Review document here is a short sentence. First, the review document is represented as bag of unigrams and bigrams feature vector representation as shown in Table 3. The probability of a review document's given certain class (positive and negative) can be calculated using the following equation:

(2) $$\begin{array}{c} P\left(\left.\text{Doc}\right|\text{Class}\right)=\prod_{i=1}^{\text{Doc}}P\left(\left.W\right|c_{i}\right), \end{array}$$

where Doc is the review document, |Doc| is the length of document, and *P*(*W*|*c*_*i*_) is the probability of a term *W* in a review document's given certain class (+ve or −ve). Table 3 shows unigrams and bigrams along with their vector representation for the corresponding review documents given in Example 1.

In order to classify a review document “*I love this movie*,” we need to determine the probabilities of all terms (unigrams and bigrams) in the review documents labeled as positive. The probability of each term given class *c*_*i*_, *P*(*w*_*k*_|*c*_*i*_), is computed as follows:

(3) $$\begin{array}{c} P\left(\left.w_{k}\right|\text{positive}\right)=\frac{n_{k}+1}{n+\left|\text{VOC}\right|}, \end{array}$$

where *n*_*k*_ is the number of times the term *w*_*k*_ occurs in positive cases and *n* is the total number of words in positive cases. |VOC|  indicates the number of unique unigrams and bigrams in the review documents. Probability of the above review document's given positive case is estimated based on probabilities of all unigrams and bigrams in the review document.

(4) $$\begin{array}{c} P\left(\text{Positive}\right)=\frac{\text{number of positive review cases }}{\text{total number of review cases}},\\ P\;\left(\left.\text{loved}\right|\text{positive}\right)=\frac{\text{number of times “loved” occurs in positive cases}+1}{\text{total number of words in positive cases}+\left|\text{VOC}\right|},\\ \left(\left.\text{this movie}\right|\text{positive}\right)=\frac{\text{number of times “this movie” occurs in positive case}+1}{\text{total number of words in positive case}+\left|\text{VOC}\right|}. \end{array}$$

So, the probability of the above review document's given positive case is expressed as follows:

(5) $$\begin{array}{c} P\left(\left.\text{review doc}\right|\text{positive}\right)=P\left(\text{Positive}\right).P\left(\left.\text{“loved”}\right|\text{Positive}\right).\;P\left(\left.\text{“loved this”}\right|\text{Positive}\right).P\left(\left.\text{“movie”}\right|\text{Positive}\right).P\left(\left.\text{“this”}\right|\text{Positive}\right).\;P\left(\left.\text{“this movie”}\right|\text{Positive}\right). \end{array}$$

Similarly, the probability of the above review document's given negative case is estimated as follows:

(6) $$\begin{array}{c} P\left(\left.\text{review doc}\right|\text{Negative}\right)=P\left(\text{Negative}\right)\text{.}P\left(\text{“loved” }|\text{ Negative}\right).P\left(\left.\text{“loved this”}\right|\text{Negative}\right).P\left(\left.\text{“movie”}\right|\text{Negative}\right).P\left(\left.\text{“this”}\right|\text{Negative}\right).P\left(\left.\text{“this movie”}\right|\text{Negative}\right). \end{array}$$

The same process is repeated for negative review document.

Based on the following equation, the review document is assigned to a class if the probability value of the review document's given class is maximized.

(7) $$\begin{array}{c} C_{NB}=\arg\max_{C_{i}\in C}P\left(C_{i}\right)\prod_{w\in \text{words}}P\left(\frac{w}{C_{i}}\right). \end{array}$$

In other words, the review document is assigned a positive class, if probability value of the review document's given class is maximized and vice versa.

### 3.4. Summarization of Reviews

The goal of this phase is to summarize the classified reviews (both positive and negative reviews). This phase comprises three steps: (1) creation of graph from classified reviews, (2) ranking of graph nodes (review sentences), and (3) selection of top rank sentences (nodes) for summary generation.

#### 3.4.1. Graph-Based Representation of Classified Reviews

The goal of this phase is to build a graph from classified reviews. First, we split the classified reviews into sentences. Next, we find semantic similarities between review sentences and construct a graph from the pairwise semantic similarities between sentences. In order to compute pairwise semantic similarities between sentences, we extract word embeddings for each word in sentences using word2vec model. We employed Google's pretrained word2vec model [57, 58] to learn word embeddings (word vectors) for each word in all sentences. The word2vec model, released by Google, is a neural network-based implementation that learns distributed vector representations of words based on continuous bag of words. The model is trained approximately on 100 billion words from Google News dataset. We leave the default word vector length to be 300 features.

In order to represent sentences as vectors, we take the mean of all word embeddings present in the vocabulary of word2vec and ignore the words not present in vocabulary. The semantic similarity between any two sentence vectors *A* and *B* is determined using cosine similarity as given in equation (8). Cosine similarity is a dot product between two vectors; it is 1 if the cosine angle between two sentence vectors is 0, and it is less than one for any other angle.

(8) $$\begin{array}{c} \text{sim}\left(A,B\right)=\cos\left(\theta \right)=\frac{A\cdot B}{\left\|\left.A\right\|\right.\left\|\left.B\right\|\right.}. \end{array}$$

**Figure 2:**
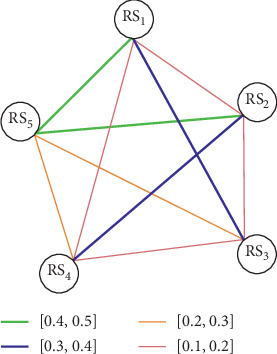
Undirected weighted graph.

Once the semantic similarity score for each pair of sentence is computed, a semantic similarity matrix *M*_*ij*_ is constructed from the similarity scores of review sentences. Next, an undirected weighted graph is built from the semantic similarity matrix constructed in previous step. The graph is created in such a way if the similarity weight sim(*A*_*i*_, *B*_*j*_) between nodes *A*_*i*_ and *B*_*j*_ (*i* *≠* *j*) is greater than 0, then a link is established between them; otherwise, no link is established. In this study, we are interested only in significant sentence similarity and thus define a similarity threshold that is empirically set to 0.5 [59]. So, a link is only established between the nodes whose similarity score lies between 0 < ∝≤0.5; else, there will be no link established between the nodes. Two nodes having similarity score greater the 0.5 are supposed to be semantically equivalent and are not added in the graph in order to avoid sentence redundancy in summary generation. The semantic similarity sim(*A*_*i*_, *B*_*j*_) between two nodes *A*_*i*_ and *B*_*j*_ (*i* ≠ *j*) is determined using equation (8).  Figure 2 depicts an undirected weighted graph. The edges displayed with different colored solid bars specify different ranges of semantic similarity weights in graph. The nodes of graph refer to the review sentences indicated by RS_*i*_ where *i* ranges from 1 to *n*.

#### 3.4.2. Ranking of Graph Nodes (Review Sentences)

Now, we formally characterize the document *D* of classified reviews; suppose *G* *=* (*V*, *E*) is an undirected weighted graph having *n* number of nodes/vertices V connected through edges *E*, which represent relationship between the classified review sentences in document set *D*. Let *V* be the set of vertices, where each vertex *v*_*i*_ in *V* denotes the classified review sentence in *D*. Assume *E* to be the set of edges with each edge *e*_*ij*_ indicating the semantic similarity weight between the two vertices *v*_*i*_ and *v*_*j*_. Next, we apply weighted graph-based ranking algorithm (WGRA) that takes into consideration the edge weights, which correspond to sentence-sentence semantic similarity. The importance score of the node/vertex *v*_*i*_ under consideration is denoted by WGRA(*v*_*i*_). The vertex/node salience score is computed from all the connected vertices (sentences) plus taking into account the salience scores of the connected vertices (sentences); formally, it is written as follows:

(9) $$\begin{array}{c} \text{WGRA}\left(v_{i}\right)=\left(1-d\right)+d*\sum_{v_{j}\in \text{In}\left(v_{i}\right)}\frac{\text{WGRA}\left(v_{j}\right)\cdot w_{ji}}{\sum_{v_{k}\in \text{Out}\left(v_{j}\right)}w_{jk}}, \end{array}$$

where *d* is damping factor and generally its value is set to 0.85 [60]. In(*v*_*i*_) are the number of vertices that are pointing to given vertex *v*_*i*_, Out(*v*_*j*_) are the number of outgoing links from the vertex *v*_*j*_, and *w*_*ji*_ represents the weight associated with the edge between nodes *v*_*i*_ and *v*_*j*_. *w*_*jk*_ represents the weights associated with outgoing links from vertex *v*_*j*_.

From the implementation point of view, weighted graph-based ranking algorithm (WGRA) starts by initializing all the graph nodes/vertices with a rank score 1. Then, the algorithm computes the number of connected nodes/vertices to the current node under consideration. Once number of connected nodes/vertices to current the node/vertex is found, the algorithm computes the importance of each connected vertex in two steps.

First, the outgoing links from given connected vertex are counted, and then the weights associated with outgoing links are aggregated. It means that ranking algorithm computes the rank score of a given node/vertex by considering the number of nodes/vertices that are connected to it as well as the salience scores of the connected vertices. Once the salience scores of the linked nodes/vertices are obtained, the WGRA uses equation (9) to compute the new ranking scores for the nodes/vertices. The algorithm continuously computes the salience scores for the nodes/vertices until convergence is attained. The convergence is achieved by the iteration/ranking algorithm, when the discrepancy between the rank scores calculated for any vertices (sentences) at two successive iterations drops under a given threshold (0.0001 in this study) [59]. After the algorithm gets converged, the rank scores attained for vertices of the graph are sorted in reverse order.

#### 3.4.3. Summary Generation

The goal of this phase is to generate summary from the classified movie review sentences. As discussed earlier, the classified review sentences (both positive and negative) are represented as graph, and the weighted graph-based ranking algorithm (WGRA) computes the rank score of each sentence in the graph. Finally, the rank scores attained for vertices (sentences) of the graph are sorted in reverse order. The next step is to choose the top ranked sentences for extractive summary generation. In this study, we chose top 20 high ranked sentences for summary.

## 4. Experimental Settings

### 4.1. Evaluation Data

The proposed approach comprises of two components: the first component is Naïve Bayes (NB) classifier, which classifies the review documents into positive and negative. The second component is semantic graph-based ranking algorithm, which performs the task of movie review summarization. In order to evaluate the first component (NB classifier), we considered document-level and sentence-level classification tasks in the domain of movie reviews.

For document-level sentiment classification task, we used two publically available movie review datasets. The first one is introduced by Pang and Lee (http://www.cs.cornell.edu/people/pabo/movie-review-data/) [61], which is a most widely used polarity dataset of 2000 movie reviews (version 2). It consists of 1000 positive movie reviews and 1000 negative reviews. Each review in the dataset is associated with binary sentiment polarity label. The second benchmark dataset is constructed by Andrew [62], which consists of 50,000 reviews from the IMDB dataset, and each movie is restricted to have no more than 30 reviews. It is comprised of movie reviews with their corresponding labels (sentiment polarity). The labeled dataset is evenly divided into 2.5 k training and 2.5 k train sets. Like previous work on polarity classification, this study also assumes high polarized reviews. The negative reviews in the dataset are scored ≤4 out of 10, while the positive reviews are scored ≥7 out of 10.

We also evaluated NB classifier on sentence-level subjectivity classification task. For this task, we used the dataset introduced by Pang and Lee [61], which contains 5000 subjective and 5000 objective sentences taken from movie review summaries and movie plot summaries, respectively. We compared NB classifier (with variations on bag of words features) with benchmark model for sentiment analysis [62], in terms of classification accuracy on the three evaluation tasks discussed above. The benchmark model used a mix unsupervised and supervised techniques to learn word vectors for capturing semantic and sentiment information.

The proposed semantic graph-based ranking algorithm for the task of movie review summarization is evaluated on 4 randomly chosen balanced subsets of classified reviews where each subset roughly contains 100 positive and 100 negative reviews. We asked 2 Ph.D. students working in the area of natural language processing to manually produce summaries for each subset of classified reviews. The performance of the proposed method is compared with state-of-the-art graph-based summarization techniques using ROUGE-1 and ROUGE-2 evaluation metrics.

### 4.2. Experimental Steps

Given the dataset, first, the preprocessing techniques are applied over the dataset to segment the dataset into sentences, tokenize the sentences into words, and remove the stop words. Word Stemming is also performed on the remaining words to stem the words to their root form. Next, document features are extracted using the BoW technique. This study uses NB ML algorithms in order to mine movie reviews. There are other commonly used supervised machine learning techniques for opinion mining like SVM and neural network; however, Naïve Bayes is chosen for classification of movie reviews based on performance accuracy.

In order to perform the movie review classification task, the Naïve Bayes classifier is used to classify the movie reviews into positive and negative. For training and testing of NB, we applied the 10-fold cross validation technique over the three balanced datasets. Two datasets, namely, PL04 and Full IMDB, as shown in Table 4, were used for document sentiment classification task, and subjectivity dataset was used for sentence-level subjectivity classification task. In this study, we used stratified 10-fold cross validation (commonly used for classification problems), in which the folds are chosen in such a way so that each fold contains roughly the same proportion of class labels.

We evaluated the classification accuracy of NB classifier with different variations on the bag of words feature sets and compared the results with the benchmark model [62] for sentiment classification as shown in Table 4. The benchmark model utilizes a mix of unsupervised and supervised techniques to learn word vectors that capture semantic term-document information as well as rich sentiment content. Line 1 in Table 4 shows that accuracy of NB classifier with only unigrams as features on smaller datasets (PL04 and Subjectivity) is superior to resulting accuracy with bigrams. However, Line 2 shows that on large IMDB dataset, the accuracy of the classifier is boosted with only bigrams as features. Line 3 shows that the accuracy of classifier is further improved when both unigrams and bigrams were used as feature set.

Line 4 shows that unigram frequency weighted with smoothed inverse document frequency (IDF) with cosine normalization has slightly degraded the classifier accuracy on smaller datasets and slightly improved the accuracy on large IMDB dataset. Line 5 shows that bigram counts weighted with IDF with cosine normalization enhanced the accuracy on all datasets. Line 6 indicates that combination of unigrams and bigrams features-count weighted with smoothed IDF with cosine normalization surpassed the benchmark model and all the variations of bag-of-words features in terms of classification accuracy on all benchmark datasets except the subjectivity dataset where the accuracy marginally fell down by 0.31% as compared to same feature set with no IDF and cosine normalization in Line 3.

Once the classifier classifies the reviews into positive and negative reviews, the proposed approach exploits semantic graph-based summarization technique to generate summary from the classified reviews. The summarization technique represents the classified review sentences through a graph and then applies the weighted graph-based ranking algorithm to rank the important graph nodes (review sentences). Finally, the top ranked review sentences constitute the summary.

For comparative evaluation, we set up two state-of-the-art graph-based summarization techniques, namely, LexRank [63] and TextRank [64]. The LexRank model represents sentences through a graph and determines their salience based on the notion of eigenvector centrality. The model builds adjacency matrix (graph representation of sentences) from connectivity matrix, which is based on intrasentence cosine similarity. The LexRank model is another graph-based ranking algorithm that creates graph representation of sentences and utilizes global information from the whole graph to decide the salience of a vertex (sentence) within a graph. The edge weight is determined from content similarity between sentences. However, our semantic graph-based approach utilizes semantic similarity between sentences to represent the edge weight.

This study employs ROUGE-1 and ROUGE-2 evaluation metrics to compare our proposed semantic graph approach with the state-of-the-art graph-based approaches for summarization, in the context of generic movie review extractive summarization task. Our proposed approach and other models perform the task of multidocument summarization since they generate summaries from multiple movie reviews (or documents).

There are many variants of ROUGE evaluation measures: ROUGE-*N* (*N* = 1, 2, 3, and 4), ROUGE-*S*, and ROUGE-*L*. But ROUGE-1 and ROUGE-2 are efficiently applied for multidocument extractive summarization task [65]. ROUGE − *N* can be defined [65] as an *n-gram* recall between a system summary and set of human (reference) summaries and is calculated as follows:

(10) $$\begin{array}{c} \text{ROUGE}-N=\frac{\sum_{S\in \left\{\text{Reference Summaries}\right\}}\sum_{{\text{gram}}_{n}\in S}{\text{Count}}_{\text{match}}\left({\text{gram}}_{n}\right)}{\sum_{S\in \left\{\text{Reference Summaries}\right\}}\sum_{{\text{gram}}_{n}\in S}\text{Count}\left({\text{gram}}_{n}\right)}, \end{array}$$

where *n* is the length of the *n-*gram, gram_*n*_ and count_match_ (*gram*_*n*_) is the maximum number of *n*-grams that simultaneously occur in a system summary and a set of human summaries.

The precision, recall, and F-measure for system summary (or candidate summary) are computed as follows:

(11) $$\begin{array}{c} \text{precision}=\frac{\text{system summary}\cap \text{human summary}}{\text{system summary}}, \end{array}$$

(12) $$\begin{array}{c} \text{recall}=\frac{\text{system summary}\cap \text{human summary}}{\text{human summary}}, \end{array}$$

(13) $$\begin{array}{c} \text{F}-\text{measure}=2*\frac{\text{precision}*\text{recall}}{\text{precision}+\text{recall}}. \end{array}$$

Tables 5 and 6 illustrate the comparative evaluation results of the proposed approach and other summarization models based on ROUGE-1 and ROUGE-2 measures, respectively. These results are achieved on randomly chosen balanced subset of classified movie reviews as discussed above. For the same subset of movie reviews, we asked 2 Ph.D. students working in area of natural language processing to manually create summaries of 20 sentences.

Referring to the ROUGE-1 results given in Table 5, our proposed graph-based technique performs better than other summarization models based on average precision, recall, and F-measure. LexRank produces better summarization results as compared to TextRank.

Similarly, based on ROUGE-2 results given in Table 6, the proposed technique still outperforms other summarization models based on average precision, recall, and F-measure. LexRank also maintained to produce better summarization results than TextRank based on ROUGE-2.

**Figure 3:**
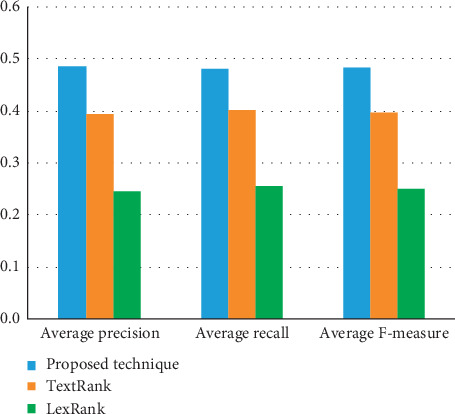
Comparison of summarization models in terms of ROUGE-1 measures.

**Figure 4:**
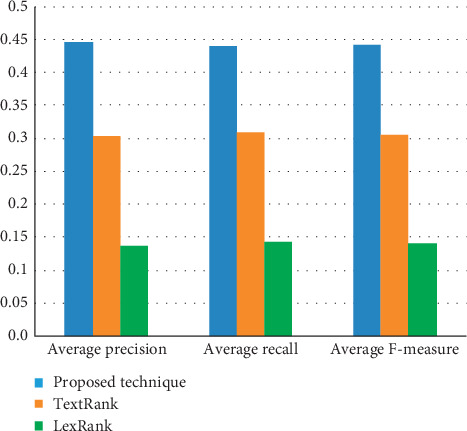
Comparison of summarization models in terms of ROUGE-2 measures.

Figures 3 and 4 visualize the summarization results of the proposed approach and other summarization models based on ROUGE-1 and ROUGE-2, respectively.

### 4.3. Discussion

This section discusses the evaluation results of classification and summarization approaches presented in previous section. First, we discuss the classification approaches for sentiment classification of movie reviews. In this study, we proposed to use NB classifier with both unigrams and bigrams as feature set for sentiment classification of movie reviews. We evaluated the classification accuracy of NB classifier with different variations on the bag-of-words feature sets in the context of three datasets that are PL04 (2000 reviews), IMDB dataset (50,000 reviews), and subjectivity dataset (1000 sentences). It can be observed from results given in Table 4 that the accuracy of NB classifier surpassed the benchmark model on IMDB and subjectivity datasets, when both unigrams and bigrams are used as features. However, the accuracy of NB on PL04 dataset was lower as compared to the benchmark model. Referring to Line 6 in Table 4, when the combination of unigrams and bigrams features-count are weighted with smoothed IDF with cosine normalization, the classification accuracy of NB classifier is further improved and surpassed the benchmark model and all the variations of bag-of-words features on all benchmark datasets except the subjectivity dataset where the accuracy marginally fell down by 0.31% as compared to same feature set with no IDF and cosine normalization in Line 3 of Table 4. It is concluded from the empirical results that combination of unigrams and bigrams as features is an effective feature set for the NB classifier as it significantly improved the classification accuracy.

Now, we discuss the summarization results of our proposed semantic graph-based approach and other state-of-the-art graph-based summarization models in the context of generic movie summarization task. The proposed approach is compared with other summarization models in terms of average precision, recall, and F-measure obtained with ROUGE-1 and ROUGE-2.

Referring to the ROUGE-1 results in Table 5, the proposed method outperformed the state-of-the-art summarization techniques and achieved improved performance in terms of precision, recall, and F-measure. LexRank stood second and TexRank stood third in terms of summarization results.

The proposed approach utilizes word2vec model to extract word vectors for all words in sentences. The feature vector for sentences is computed by averaging all the word vectors in each sentence. The cosine similarity of feature vector representation of sentences will capture semantically related sentences. So, it assists the graph ranking algorithm in selection of high ranked review sentences (nodes) by taking its votes from other review sentences (nodes) that are semantically related to it. The experimental outcomes justify that proposed semantic graph-based ranking algorithm embedded with semantic similarity considerably improved the summarization results.

In order to validate the results, we also carried out statistical significance tests (*T*-tests) to show the enhancement of our proposed approach with other state-of-the-art summarization models. The paired-sample *T*-test procedure was used to compare the means of two results that represent the same test group and obtained low significance values of 0.039, 0.030, and 0.029 for average precision, recall, and F-measure, respectively. The low significance values for the *T*-test (typically less than 0.05) show that there is a significant difference between the results of the proposed approach and other summarization models.

## 5. Conclusion and Future Work

Movie review mining and summarization is a challenging task, and this study sets a new direction in movie review summarization. Few research efforts have been made in the domain of movie reviews. We proposed an approach that classifies and summarizes the movie reviews using the ML technique and graph-based ranking. The proposed approach is general and is applicable to any domain by just providing the training data of that specific domain.

In the context of movie review sentiment classification, we found that Naïve Bayes classifier performed very well as compared to the benchmark method when both unigrams and bigrams were used as features. The performance of the classifier was further improved when the frequency of features (unigrams and bigrams) was weighted with IDF.

Finally, we used the semantic graph-based approach to summarize the classified movies reviews in order to provide a gist of gigantic amount of movie reviews. From the empirical results, we concluded that the proposed approach performs better than other state-of-the-art summarization models.

In future, we plan to apply deep learning models to generate abstractive summary from movie reviews. Furthermore, we would like to extend our technique to other domains and examine the effectiveness of the proposed technique.

## Figures and Tables

**Table 1:** BoW vector space model for unigrams.

Review documents	Acting	Good	Great	Hated	Loved	Movie	This	Class
Review Doc1	0	0	0	0	1	1	1	+ve
Review Doc2	0	0	0	1	0	1	1	−ve
Review Doc3	1	1	1	0	0	1	0	+ve

**Table 2:** Bag of bigram vector space model.

Review documents	Acting good	Good movie	Great acting	Hated this	Loved this	This movie	Class
Review Doc1	0	0	0	0	1	1	+ve
Review Doc2	0	0	0	1	0	1	−ve
Review Doc3	1	1	1	0	0	0	+ve

**Table 3:** Vector space model of bag of unigrams and bigrams.

Review documents	Acting	Acting good	Good	Good movie	…	Loved this	Movie	This	This movie	Class

Review Doc1	0	0	0	0	…	1	1	1	1	+ve
Review Doc2	0	0	0	0	…	0	1	1	1	−ve
Review Doc3	1	1	1	1	…	0	1	0	0	+ve

**Table 4:** Movie review classification accuracy on three tasks.

	Features	PL04	Full IMDB	Subjectivity
1	Unigrams with NB	81.5	86.66	90.75
2	Bigrams with NB	77.7	88.29	76.03
3	Unigrams + bigrams with NB	82.4	88.91	**91.22**
4	Unigram frequency + smoothed IDF + cosine normalization	82.1	87.36	90.7
5	Bigram frequency + smoothed IDF + cosine normalization	81.15	88.31	76.72
6	Unigrams + bigrams + smoothed IDF + cosine normalization	**83.7**	**89.28**	90.91
10	Benchmark model [62]	88.90	88.89	88.13

PL04 refers to the collection of 2000 movie reviews often used as benchmark dataset for sentiment classification [61], Full IMDB dataset is a collection of 50,000 reviews, and sentence subjectivity dataset is a collection of 1000 movie reviews [61].

**Table 5:** Comparison of the proposed summarization technique with other summarization models based on different measures obtained with ROUGE-1.

Techniques	Average precision	Average recall	Average F-measure
Proposed technique	**0.48485**	**0.47925**	**0.482**
LexRank [63]	0.39215	0.3997	0.3959
TextRank [64]	0.24515	0.25535	0.25015

**Table 6:** Comparison of the proposed summarization technique with other summarization models based on different measures obtained with ROUGE-2.

Techniques	Average precision	Average recall	Average F-measure
Proposed technique	**0.4439**	**0.4388**	**0.44135**
LexRank [63]	0.30195	0.30805	0.305
TextRank [64]	0.13595	0.14215	0.139

## Data Availability

The data used to support the findings of this study are available from the following website: https://www.imdb.com/.
